# Educational status, testosterone replacement, and intelligence outcomes in Klinefelter syndrome

**DOI:** 10.1590/1980-5764-DN-2021-0049

**Published:** 2022

**Authors:** Luciane Simonetti, Magnus Regios Dias da Silva, Claudia Berlim de Mello

**Affiliations:** 1 Universidade Federal de São Paulo, Departamento de Medicina, Divisão de Endocrinologia, São Paulo SP, Brazil.; 2 Universidade Federal de São Paulo, Departamento de Psicobiologia, São Paulo SP, Brazil.

**Keywords:** Klinefelter Syndrome, Phenotype, Intelligence, Testosterone, Educational Status, Síndrome de Klinefelter, Fenótipo, Inteligência, Testosterona, Escolaridade

## Abstract

Most male hypergonadotropic hypogonadism associated with infertility can be attributed to a single genetic condition such as Klinefelter syndrome (KS). This disease's wide phenotypic variability is frequently associated with mosaic 47,XXY lineages and testosterone replacement. Early diagnosis and treatment have been associated with better cognitive and intellectual outcomes, but the scope of this influence requires further investigation.

**Objective:**

This study aimed to investigate the intelligence profile of a cohort of patients with KS, considering the influence of educational level and clinical variables.

**Methods:**

Twenty-nine (9–65 years) individuals were submitted to the measures of intelligence quotient (IQ) (Wechsler's Scales) and adaptive behavior (Vineland-II). Linear regression analysis included the participants’ educational level and clinical variables (i.e., comorbidities and use of testosterone) as predictors and intellectual performance and adaptive behavior as outcomes.

**Results:**

Scores varied from intellectual deficiency to average ranges (82.5+15.8). There were significant differences between adult's and children's IQ and between verbal and nonverbal indexes. The level of education predicted both IQ and adaptive behavior. Testosterone replacement therapy and absence of seizures predicted only adaptive behavior.

**Conclusions:**

The level of education and hormonal therapy can be selectively implicated in the intellectual variability in KS.

## RESUMO.

A maioria dos casos de hipogonadismo hipergonadotrófico masculino associado à infertilidade pode ser atribuída a uma única condição genética — a síndrome de Klinefelter (KS). A ampla variabilidade fenotípica dessa doença está frequentemente associada a linhagens de mosaico 47,XXY e também à reposição de testosterona. O diagnóstico e o tratamento precoces têm sido associados a melhores desfechos em termos de cognição e inteligência, mas o escopo dessa influência requer maior investigação.

**Objetivo:**

Este estudo investigou o perfil de inteligência de uma coorte de pacientes com KS, considerando a influência do nível educacional e das variáveis clínicas.

**Métodos:**

Vinte e nove indivíduos (9–65 anos) foram submetidos a medidas de quociente de inteligência (escalas Wechsler) e de comportamento adaptativo (escala Vineland-II). A análise de regressão linear considerou o nível educacional dos participantes e variáveis clínicas (comorbidades, uso de testosterona) como preditores e desempenho intelectual e comportamento adaptativo como desfechos.

**Resultados:**

Os resultados mostraram escores que variaram de deficiência intelectual à faixa média (82,5+15,8). Houve diferenças significativas entre os quocientes de inteligência de adultos e crianças e entre os índices verbais e não verbais. O nível educacional influenciou tanto o quociente de inteligência quanto o comportamento adaptativo. A terapia de reposição de testosterona e a ausência de convulsões influenciaram apenas o comportamento adaptativo. .

**Conclusões:**

Sendo assim, nível educacional e terapia hormonal podem estar seletivamente implicados na variabilidade intelectual na KS.

## INTRODUCTION

Klinefelter syndrome (KS) is a male X-chromosome aneuploidy, resulting in 47,XXY karyotype^1,2^. It is the most frequent cause of hypergonadotropic hypogonadism and the main genetic cause of male infertility^3,4^, and it occurs in approximately 1 in 650 males^5^. The diagnosis usually occurs in adulthood due to infertility, when karyotyping test confirms X-chromosome polysomy 47,XXY, 48,XXXY, and 49,XXXXY or its mosaic forms 47,XXY/46,XY and 47,XXY/48,XXXY. The most well-known characteristics, alongside infertility, are gynecomastia, tall height, smaller testes and penis, azoospermia, and low levels of testosterone^6,7^.

Genotypic and phenotypic variability in KS is the subject of many studies^8,9^. Mosaicism and hormonal dysfunctions are related to a wide range of phenotypic manifestations^10^. In addition to infertility, individuals may present late-onset disorders, such as diabetes mellitus, obesity, metabolic and cardiovascular abnormalities, and epilepsy^7,11^. Cognitive, social, and language deficits have also been reported, but the overall intelligence quotient (IQ) is usually at average levels^12-14^. There is an increased risk for learning disabilities, poor adaptive functioning, internalizing symptoms (i.e., anxiety and depression), and attention-deficit hyperactivity disorder^14^. Impairments in executive functions, such as working memory and cognitive flexibility, may be present independently of neurodevelopmental disorders, probably due to the effects of hormonal dysfunctions on brain maturation^15,16^. The phenotypic heterogeneity in KS incurs diagnosis and treatment delays. There is evidence that early hormonal therapy minimizes cardiovascular and metabolic dysfunctions^11^ and has positive effects on cognitive and behavioral functioning^17,18^.

Since delayed treatment and higher morbidity in KS have been associated with low socioeconomic status^3,19^, analysis of influences of clinical and educational variables may favor a better understanding of intellectual variability. This study aimed to investigate the IQ and adaptive behavior of a sample of Brazilian individuals with KS, considering the association of educational and clinical variables. We hypothesized that higher educational levels and testosterone replacement would be associated with better outcomes. Therefore, this study is of special relevance since results may contribute to both the understanding of phenotypic characteristics of KS and the clinical follow-up of diagnosed individuals.

## METHODS

### Participants and study design

This study followed a cross-sectional design for clinical studies, with a report on series of patients with an outcome of interest. A total of 29 individuals with KS (28 with 47,XXY karyotype and 1 with 48,XXXY karyotype) aged from 9 to 65 years were enrolled in the study. All were recruited from the Development Ambulatory of the Endocrinology Division of the Medical School of the Universidade Federal de São Paulo, Brazil. A clinical endocrinologist examined all participants. The diagnosis was based on a thorough investigation, involving physical examination, assessment of hormone levels, and karyotyping. Data regarding hormone levels were obtained from medical records. Testosterone levels were measured by using liquid chromatography-tandem mass spectrometry in the same laboratory.

Information concerning clinical and socioenvironmental characteristics of participants was obtained by means of interviews with the adult participants or with the children's and adolescents’ caregivers. Clinical variables included the history of delay in language development, seizures, mood oscillations, and testosterone replacement. Language delays have been associated with long-term consequences for typically developing children and are present in most neurodevelopmental disorders^20^. Socioenvironmental variables included age and educational level of participants or of their caregivers. We considered educational level as years spent at school.

This study was in accordance with all ethical norms and approved by the Research Ethics Committee of Universidade Federal de São Paulo (1180/2016). All adult subjects signed a consent form, as well as the legal guardians of those under the age of 18. All children and adolescents also signed the assent forms. All assessment procedures were executed from April 2017 to December 2018.

### Intellectual assessment procedures

Intellectual assessment focused on IQ and adaptive behavior. IQ was assessed with the Brazilian versions of the Wechsler Adult Intelligence Scale (WAIS-III)^21^ and the Wechsler Intelligence Scale for Children (WISC-IV)^22^. The Vineland-II Adaptive Behavior Scale (VABS-II) was used for both age groups^23^. Assessment occurred individually in two sessions of 90-min each.

The WAIS-III comprises 13 core subtests and the WISC-IV comprises 10 subtests. Since some of the subtests differ in versions, we included the supplementary *Picture Completion*, *Arithmetic* and *Information* from the WISC-IV and *Picture Arrangement* from the WISC-III^24^ to assure equivalent measures for all age groups. The verbal indexes, such as Verbal Intelligence Quotient (VIQ; WAIS-III) and Verbal Comprehension Index (VCI; WISC-IV), and the nonverbal indexes, such as Performance IQ (PIQ; WAIS-III) and Perceptual Reasoning Index (PRI; WISC-IV), were compared. The VABS-II covers four domains, namely, communication, daily living skills, socialization, and motor skills. The overall scores of both scales are expressed as standard scores (mean=100, standard deviation [SD]=15).

### Statistical analysis

Descriptive analysis was used for sociodemographic and clinical characteristics. To investigate the associations among educational, clinical, and intellectual variables, simple and exploratory linear regression models were used. The adequacy of models was evaluated by the normality of residues observed in qqplot graphs. RStudio^©^ and GraphPad Prism^©^ software were used for the analysis. The level of significance was set to be 0.05%.

## RESULTS

Educational and clinical data are presented in Table 1. The sample is composed of 6 children and adolescents aged 9–17 years (M_age_=13.17, SD=3.4), and 23 adults aged from 18 to 65 years (M_age_=35.3, SD=14.1). The average education level of participants and their caretakers was 6.9 (SD=4.5) years and 5.9 (SD=4.3) years, respectively. Most had attended or were still attending public schools; 34.5% were illiterate. Mood oscillations were reported in 79% of cases and history of language delay in 45%. A proportion of 62% was on testosterone replacement treatment.

**Table 1:** Sociodemographic and clinical data of participants (n=29).

Sociodemographic		Mean (SD)	%
Age (years)		30.7 (15.5)	
Educational level (years)		6.9 (4.5)	
Mother's education level (years)		5.9 (4.3)	
School			
	Public	27	93.1
	Private	2	0.7
Literate		19	65.5
Illiterate		10	34.5
Clinical			
Speech delay			
	Yes	13	44.8
	No	8	27.6
	Unknown	8	27.6
Seizures			
	Yes	6	20.7
	No	23	79.3
Testosterone replacement			
	Yes	18	62.1
	No	11	37.9
Mood oscillation			
	Yes	23	79.3
	No	6	20.7

SD: standard deviation.

Intellectual performance data are presented in Table 2.

**Table 2:** Intelligence data of participants.

Intellectual assessment		Mean (SD)	Median	Min–Max
WAIS-III (n=25)				
	FSIQ	86.2 (13.1)	83	68–111
	PIQ	92.6 (16.7)	85	70–122
	VIQ	82.0 (10.7)	80	66–102
WISC-IV (n=4)				
	FSIQ	59.5 (11.3)	57.5	48–75
	POI	71.7 (18.8)	89	57–86
	VCI	51.0 (7.1)	77	45–61
	All patients (FSIQ)	82.5 (15.8)	81	48–111
Subtests				
	Figure completion	8.4 (4.0)	8	1–15
	Block design	8.7 (3.0)	8	3–15
	Picture arrangementα	8.5 (3.9)	9	1–18
	Matrix reasoning	8.3 (3.1)	8	3–16
	Coding	8.4 (2.8)	9	3–15
	Digit symbol	9.7 (4.0)	9	4–19
	Figurative concepts&	4.5 (1.9)	4	3–7
	Vocabulary	4.4 (2.5)	4	1–9
	Similarities	6.6 (3.0)	6	1–13
	Comprehension	5.0 (2.1)	5	1–9
	Information	7.3 (2.6)	7	1–12
	Arithmetic	7.1 (2.6)	7	1–11
	Digit span	7.2 (3.6)	7	1–17
	Letter-number sequencing	6.7 (3.1)	6	2–15
VABS-II				
	Adaptive behavior composite£	72.3 (23.6)	78	20–112
	Communication	62.4 (26.6)	67	21–107
	Daily living skills	82.9 (22.5)	87	31–116
	Socialization	79.0 (26.8)	85	20–121
	Motor skills	95.7 (18.7)	94	40–121

SD: standard deviation; WAIS-III: Wechsler Adult Intelligence Scale; FSIQ: Full Scale; PIQ: Performance Intelligence; VIQ: Verbal Intelligence; POI: Perceptual Organization Index; VCI: Verbal Comprehension Index; WISC-IV: Wechsler Intelligence Scale for Children (WISC-IV); VABS-II: The Vineland-II Adaptive Behavior Scale;

& WISC-IV's figurative concepts subtest applied only for children/adolescents;

£ Vineland-II Adaptive Behavior Scales: average ranging from 85 to 115.

IQ varied from intellectual disability to average levels. Important differences (26.7 points) between the mean IQ obtained by children/adolescents and adults (see Table 2) were detected. For both age ranges, a better performance was detected in nonverbal indices (PIQ: M=92.6, SD=16.7 and PRI: M=71.7, SD=18.8). Children/adolescents showed intellectual disability and a significant discrepancy (20.7 points) between verbal and nonverbal indices. Of 13 participants with IQs in intellectual disability or borderline levels, 7 presented neurological (e.g., epilepsy and episodic ataxia), psychiatric (e.g., schizophrenia and autism), skeletal, or cardiac dysfunctions.

The highest scores were observed in Digit Symbol subtest, which requires visuomotor coordination and processing speed, and the lowest in Vocabulary and Comprehension, which assesses concept formation and understanding of social situations. The overall mean score for adaptive behaviors was moderately below normative parameters (72.3; SD=23.6). Lower scores were observed in the Communication (i.e., receptive, expressive, and written) and Motor domains.

Linear regression analysis revealed higher IQ (B=22.67, p<0.001) and adaptive index (B=18.67, p=0.012) in participants with more than 10 years of education when compared with those up to 5 years. The mother's education level showed no effect for both measures. Full IQ was higher among participants with no history of seizures (B=-14.19, p=0.048) and under hormone replacement treatment (B=12.22, p=0.041) (Tables 3 and 4).

**Table 3:** Linear regression applying educational level and clinical variables for predicting intelligence quotient in Klinefelter syndrome cohort.

Educational		Descriptive	Regression			
		Mean [95%CI]	B	SE	95%CI	p-value
Participant's education (years)						
	1–5	78.92 [71.83; 64.74]	Ref.			
	6–9	79.80 [98.54; 61.06]	7.97	6.51	−5.41; 21.34	0.231
	>10	94.50 [102.17; 86.82]	22.67	4.99	12.41; 32.92	<0.001**
Mother's education level (years)						
	1–5	79.69 [88.68; 70.69]	Ref.			
	6–9	87.75 [110.53; 64.97]	8.06	9.09	−10.67; 26.79	0.384
	>10	84.37 [97.52; 71.22]	4.69	7.04	−9.82; 19.20	0.512
Clinical						
Speech delay						
	No	87.87 [102.36; 73.38]	Ref.			
	Yes	78.00 [87.55; 68.44]	-9.87	7.09	-24.46; 4.71	0.176
	Unknown	84.75 [96.49 73.02]	-3.12	7.89	-19.35; 13.10	0.695
Seizures						
	No	85.52 [92.49; 78.55]	Ref.			
	Yes	71.33 [80.08; 62.59]	-14.19	6.87	-28.28; −0.10	0.048*
Receiving testosterone						
	No	75.00 [86.29; 63.71]	Ref.			
	Yes	87.22 [94.01; 80.43]	12.22	5.70	0.52; 23.92	0.041*
Mood oscillations						
	No	76.83 [92.19; 61.47]	Ref.			
	Yes	84.08 [91.04; 77.13]	7.25	7.26	-7.63; 22.14	0.326

* p≤0.01;

** p≤0.05.

**Table 4:** Linear regression applying educational level and clinical variables for predicting adaptive behavior in Klinefelter syndrome cohort.

Educational		Descriptive	Regression			
		Mean [95%CI]	B	SE	95%CI	p-value
Participant's education (years)						
	1–5	59.33 [74.32; 44.34]	Ref.			
	6–9	78.00 [99.84; 56.15]	18.67	11.47	-4.92; 42.26	0.115
	>10	83.00 [96.17; 69.82]	23.67	8.80	5.57; 41.76	0.012*
Mother's education level (years)						
	1–5	69.62 [82.00; 57.24]	Ref.			
	6–9	90.75 [114.47; 67.03]	21.12	12.63	-4.89; 47.14	0.107
	>10	64.75 [84.74; 44.76]	-4.87	9.79	-25.03; 15.28	0.623
**Clinical**						
Speech delay						
	No	66.50 [92.98; 40.02]	Ref.			
	Yes	72.84 [85.44; 60.24]	6.346	10.85	-15.96; 28.65	0.564
	Unknown	77.37 [94.45; 60.29]	10.87	12.07	-13.94; 35.69	0.376
Seizures						
	No	76.30 [85.36; 67.24]	Ref.			
	Yes	57.17 [87.74; 26.60]	-19.138	10.40	-40.48; 2.20	0.077
Testosterone replacement						
	No	72.54 [82.61; 62.48]	Ref.			
	Yes	72.22 [86.18; 58.26]	-0.32	9.21	-19.22; 18.57	0.97
Mood oscillations						
	No	73.83 [106.38; 41.28]	Ref.			
	Yes	71.96 [81.54; 62.37]	-1.88	11.03	-24.50; 20.75	0.866

Bold data are statistically significant;

* p≤0.01;

B: unstandardized regression coefficient; SE: standard error.

**Figure 1:**
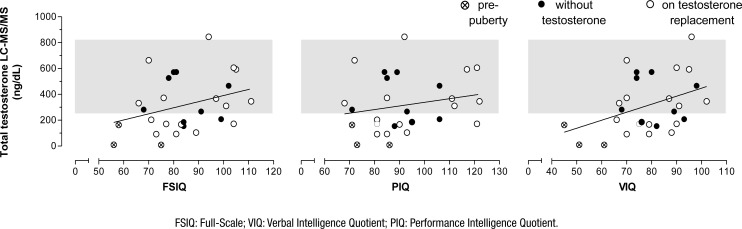
Graphic representation of simple linear regression and correlation between total testosterone and intellectual performance (28 participants). Full-Scale (FSIQ), Verbal (VIQ), and Performance (PIQ) Intelligence Quotient in Klinefelter syndrome cohort. Gray shaded area represents the reference range for total testosterone measured by liquid chromatography tandem mass spectrometry (241–827 ng/dL). Pearson's correlation r=0.4016, p=0.0341 for VIQ.

A Pearson correlation analyzed the associations between testosterone levels and intellectual scores in 28 participants because of missing data in medical records (Figure 1). All datasets followed the Gaussian distribution by using Kolmogorov-Smirnov test. Statistical significance was found for VIQ.

## DISCUSSION

This study provides information concerning intellectual variability in KS, which can contribute to clinical management as well as to further research purposes regarding phenotypic characteristics. As expected, having more years of schooling and being on testosterone replacement therapy predicted outcomes in our cohort of 29 patients with different ages. Moreover, clinical variables influenced only adaptive behavior. To the best of our knowledge, this was the first study to assess intelligence outcomes in KS, considering IQ and adaptive behavior measures, as well as environmental and clinical variable influences.

A total of 13 participants presented IQ in intellectual disability or borderline levels, including 4 participants aged <16 years. Reports of intellectual deficits in KS are not usual, regardless of age. For instance, in a sample of 47 patients diagnosed with extra X-chromosome and age ranging from 6 to 20 years, the IQs were in average levels^13^.

The lower IQ scores among our children/adolescents could be explained by the sample characteristics, since there were only four participants aged <16 years, but diagnostic conditions shall also be considered. Since KS is identified mainly in adulthood due to infertility, diagnosis in early ages is uncommon. Most of the prepubertal children treated in our endocrinology service were referred by pediatricians because of complaints of neurodevelopment delays, learning disabilities, or clinical conditions such as epilepsy and psychiatric disorders. In this study, all children and adolescents presented one or more of these clinical conditions, which can affect intellectual development. Four adult patients also reported clinical conditions, specifically seizures. Two of them were on anticonvulsant medication. The incidence of seizures in KS is estimated to be 5%^2,25^ and has been associated with mortality^1^ and low IQ^25^.

Verbal indexes were lower than nonverbal, in accordance with previous findings^12,26-28^, although the difference is not always statistically significant^29-31^. Worse performances in neuropsychological tasks that demand verbal processing in comparison with nonverbal have been frequently reported^32,33^. Interestingly, Vocabulary and Comprehension subtests’ scores were the lowest in our KS cohort, regardless of age. A qualitative analysis of participant's answers indicates a poor understanding of social situations, shortcomings in practical reasoning, restricted vocabulary, and a concrete narrative. Low performance in Comprehension was reported previously^12^. The low scores seem to reflect everyday life problems that KS individuals struggle with. The low adaptive scores express the reduced levels of functionality and autonomy. Weaknesses in practical intelligence and poor understanding of social situations are possibly related to social cognition deficits usually reported in patients^34,35^.

Our results also indicate that intelligence variation was better explained by individual's educational status rather than their caregivers’. The influence of environmental factors on the expression of intelligence skills is well known. In typical development, verbal IQ variation is moderated by parental education^36^. In genetic conditions, this influence is a matter of controversy. For example, in a large cohort (n=1,909) of sibling pairs of adolescents with Fragile X syndrome, inheritability influenced more verbal IQ variation than environmental factors among participants with high-educated parents^37^. Significantly combined influences were observed only among those with parents with <12 years of education. In a study comparing 8- to 18-year-old American and Dutch KS boys, significantly higher VIQ and PIQ were found in the former^32^. Parental education, time of diagnosis (prenatal/postnatal), and testosterone replacement were associated with differences, indicating the impact of socioeconomic factors in accessing medical treatment.

This study has limitations since all participants were recruited from a single outpatient clinic where usually more symptomatic patients are addressed. However, it innovates by emphasizing clinical and educational influences on neurocognitive phenotype, this way providing useful information for multidisciplinary approaches. The earlier signs of immature adaptive behavior or learning disabilities among children and adolescents with 47,XXY are identified, the sooner they may be submitted to interventions for improving academic and social skills. Results may contribute for a better understanding of phenotypic variability in KS.

Our results indicate that educational level and clinical variables, such as testosterone replacement and history of seizures, can be implicated in intellectual heterogeneity in KS. Higher education levels seem to influence adaptive behavior development. Further studies may elucidate the impact of testosterone replacement on cognition and behavior in life span.
